# Development of an LC–MS/MS method for the determination of five psychoactive drugs in postmortem urine by optimization of enzymatic hydrolysis of glucuronide conjugates

**DOI:** 10.1007/s11419-024-00685-1

**Published:** 2024-04-01

**Authors:** Tomohito Matsuo, Tadashi Ogawa, Masae Iwai, Katsutoshi Kubo, Fumio Kondo, Hiroshi Seno

**Affiliations:** 1 Department of Legal Medicine, Aichi Medical University School of Medicine, 1-1 Yazakokarimata, Nagakute, Aichi 480-1195 Japan; 2 Poison Analysis Center, Aichi Medical University School of Medicine, 1-1 Yazakokarimata, Nagakute, Aichi 480-1195 Japan; 3 Department of Oral Pathology/Forensic Odontology, Aichi Gakuin University School of Dentistry, 1-100 Kusumoto-Cho, Chikusa-Ku, Nagoya, Aichi 464-8650 Japan; 4 Department of Biomedical Sciences, Chubu University College of Life and Health Sciences, 1200 Matsumoto‑cho, Kasugai, Aichi 487‑8501 Japan

**Keywords:** Psychoactive drugs, Glucuronide conjugates, β-glucuronidase, Hydrolysis, Postmortem urine, LC–MS/MS

## Abstract

**Purpose:**

Toxicological analyses of biological samples play important roles in forensic and clinical investigations. Ingested drugs are excreted in urine as conjugates with endogenous substances such as glucuronic acid; hydrolyzing these conjugates improves the determination of target drugs by liquid chromatography–tandem mass spectrometry (LC–MS/MS). In this study, we sought to improve the enzymatic hydrolysis of glucuronide conjugates of five psychoactive drugs (11-nor-9-carboxy-Δ^9^-tetrahydrocannabinol, oxazepam, lorazepam, temazepam, and amitriptyline).

**Methods:**

The efficiency of enzymatic hydrolysis of glucuronide conjugates in urine was optimized by varying temperature, enzyme volume, and reaction time. The hydrolysis was performed directly on extraction columns. This analysis method using LC–MS/MS was applied to forensic autopsy samples after thorough validation.

**Results:**

We found that the recombinant β-glucuronidase B-One® quantitatively hydrolyzed these conjugates within 3 min at room temperature directly on extraction columns. This on-column method saved time and eliminated the loss of valuable samples during transfer to the extraction column. LC–MS/MS-based calibration curves processed with this method showed good linearity, with *r*^2^ values exceeding 0.998. The intra- and inter-day accuracies and precisions of the method were 93.0–109.7% and 0.8–8.8%, respectively. The recovery efficiencies were in the range of 56.1–104.5%. Matrix effects were between 78.9 and 126.9%.

**Conclusions:**

We have established an LC–MS/MS method for five psychoactive drugs in urine after enzymatic hydrolysis of glucuronide conjugates directly on extraction columns. The method was successfully applied to forensic autopsy samples. The established method will have broad applications, including forensic and clinical toxicological investigations.

## Introduction

As the variety of drugs of abuse has continued to expand, toxicological analyses have become increasingly important. Liquid chromatography–tandem mass spectrometry (LC–MS/MS), which is highly specific and sensitive and is capable of the simultaneous measurement of multiple analytes, is frequently used for these toxicological analyses. However, since ingested drugs are excreted in the urine as conjugates with glucuronic acid and other endogenous substances during phase II metabolism, only minute amounts of the free or unaltered drugs and metabolites are typically present in urine, decreasing the sensitivity and reliability of toxicological analyses. Several methods exist for the direct detection of glucuronide conjugates, but multiple issues, such as the high cost of standard glucuronide substances and their deuterated internal standards (ISs), have hampered their application. Therefore, to accurately measure urinary drug concentrations, it is preferred to analyze the samples both before and after hydrolysis [1, 2].

Enzymes can be efficient at hydrolyzing glucuronides, and they have been shown to improve the sensitivity of analysis of these compounds. For example, several studies have reported the analysis of psychoactive drugs in urine after hydrolysis using recombinant β-glucuronidases. Morris et al. reported maximum hydrolysis of the glucuronide controls of oxazepam, lorazepam and temazepam was observed at 55 °C for 45 min at pH 6.8 (mean analyte recovery ≥ 94%) and at room temperature for 5 min at pH 6.8 (mean analyte recovery ≥ 94% for oxazepam and lorazepam and ≥ 80% for temazepam) [3]. Sitasuwan et al. observed that recombinant β-glucuronidase gave 100% recovery of amitriptyline glucuronide and > 80% of temazepam glucuronide even without incubation at pH 7.4 and achieved complete hydrolysis of temazepam glucuronide within 15 min at 58 °C [4]. Kang et al. reported recombinant β-glucuronidase exhibited complete hydrolysis of glucuronide conjugates of lorazepam, oxazepam and temazepam at 55 °C within 15 min at pH 6.8 [5]. Mastrianni et al. observed that recombinant β-glucuronidase achieved 99.3% hydrolysis of amitriptyline glucuronide at 60 °C in 30 min of incubation at pH 7.4 [6]. However, the use of enzymes is also accompanied by important disadvantages, including the need to heat reaction mixtures to 55–60 °C. In addition, the enzymatic hydrolysis of 11-nor-9-carboxy-Δ^9^-tetrahydrocannabinol (THC-COOH) [7, 8], benzodiazepines [9, 10] and amitriptyline [6, 11] from biological matrices requires long reaction times of several hours. In particular, glucuronides linked to quaternary ammonium compounds, such as amitriptyline, have been reported to be challenging to hydrolyze with β-glucuronidase extracted from mollusks [12, 13]. Furthermore, impurities in β-glucuronidase preparations have been reported to further transform benzodiazepines [9, 10].

Recently, recombinant β-glucuronidase preparations that are usable at room temperature have been developed, and these enzymes offer a substantial improvement in hydrolysis procedures. In this study, we investigated the use of two of these recombinant β-glucuronidases, B-One® and IMCSzyme® RT, as well as β-glucuronidase from *Patella vulgata*, in the hydrolysis of glucuronide conjugates of five psychoactive drugs (THC-COOH, oxazepam, lorazepam, temazepam, and amitriptyline). The hydrolyzed samples were subjected to extraction with ISOLUTE® HYDRO DME + columns, and the analytes were ultimately detected by LC–MS/MS.

## Materials and methods

### Chemicals and materials

The standards amitriptyline, amitriptyline *N*-β-D-glucuronide, lorazepam, lorazepam glucuronide, oxazepam, oxazepam glucuronide, temazepam, temazepam glucuronide, THC-COOH and THC-COOH glucuronide and the ISs amitriptyline-*d*_3_, lorazepam-*d*_4_, oxazepam-*d*_5_, temazepam-*d*_5,_ and THC-COOH-*d*_9_ were purchased from Cerilliant (Round Rock, TX, USA). Stock solutions were prepared at concentrations of 100 µg/mL in methanol and stored at − 30 °C until analysis. LC–MS-grade acetonitrile, formic acid, and methanol were purchased from Fujifilm Wako Pure Chemicals (Osaka, Japan). Native β-glucuronidase isolated from *P. vulgata* (100,000–200,000 modified Fishman units/mL (U/mL)) and potassium acetate buffer solution (100 mM, pH 5.0) were obtained from Sigma Aldrich (St. Louis, MO, USA). IMCSzyme® RT (> 200,000 U/mL) was purchased from Integrated Micro-Chromatography Systems (Irma, SC, USA). B-One® (≥ 144,000 U/mL) was purchased from Kura® Biotech (Parcelación Neumann, Puerto Varas, Chile). ISOLUTE® HYDRO DME + columns were obtained from Biotage (Uppsala, Sweden). Laboratory distilled water was purified using a Direct-Q UV 3 system (Millipore, Molsheim, France). Other common chemicals used were of the highest purity commercially available. Normal human urine (NHU) used for the calibrators and quality controls (QCs) was obtained from healthy volunteers with informed consent.

### Enzyme optimization

The enzymes were diluted according to the manufacturer's recommendations at pH 5.0 for *P. vulgata* β-glucuronidase, pH 5.5 for IMCSzyme® RT, and pH 6.8 for B-One®. To perform optimization experiments, each glucuronide conjugate in methanol was spiked into blank human urine at concentrations of 500 ng/mL. Typical reaction mixtures consisted of 50 µL of spiked urine, 50 µL of β-glucuronidase solution and 10 µL of IS in methanol (500 ng/mL for amitriptyline-*d*_3_ and temazepam-*d*_5_; and 1000 ng/mL for lorazepam-*d*_4_, oxazepam-*d*_5_ and THC-COOH-*d*_9_). The efficiency of enzymatic hydrolysis was optimized by varying several parameters, including temperature (25, 37, 50, 60 or 70 °C), enzyme volume (0–7200 U for B-One® and 0–10,000 U for IMCSzyme® RT), and reaction time (1–30 min). Hydrolysis efficiency was calculated as % hydrolysis by dividing the total free analyte after hydrolysis by the theoretical concentration value of the free analyte if the glucuronide conjugate was completely hydrolyzed.

### Sample extraction

Compounds extracted with ISOLUTE® HYDRO DME + columns were eluted via centrifugation rather than with positive pressure- or vacuum-based systems; the centrifugation approach proved advantageous in terms of simplicity of handling, as previously reported [14]. The hydrolyzed sample was applied to an extraction column, and a mixture containing 10 µL of 1 M HCl and 600 µL of acetonitrile was added. The column was centrifuged at 2000 g for 5 min, and the solution was evaporated using a centrifugal evaporator (CVE-2000; Tokyo Rikakikai, Tokyo, Japan). The residue was reconstituted in 100 µL of methanol and analyzed by LC–MS/MS.

### LC–MS/MS

All analyses were performed on a Nexera X3 and LCMS-8045 system (Shimazu, Kyoto, Japan). For separation, a Kinetex XB-C 18 column (2.1 × 100 mm, 2.6 µm; Phenomenex, Torrance, CA, USA) was used at 40 °C. The mobile phase consisted of a gradient of solvent A (an aqueous solution of 0.1% formic acid and 10 mM ammonium formate) and solvent B (a methanolic solution of 0.1% formic acid and 10 mM ammonium formate) delivered at 0.5 mL/min. The composition of the mobile phase began at 30% B and linearly increased over 4.0 min to 95% B, where it was maintained for 2.0 min. The mobile phase was returned to 30% B over 0.01 min and held at 30% B for 1.0 min to equilibrate the column for the next sample.

The MS/MS was operated with an electrospray ionization source in the positive ionization mode. Ionization source conditions were as follows: nebulizer gas flow rate, 3.0 L/min; drying gas flow rate, 10 L/min; desolvation line temperature, 250 °C; heating block temperature, 400 °C; and collision gas pressure, 230 kPa. High-purity argon and nitrogen were used as collision and nebulizer gases, respectively. Quantification was performed by selected reaction monitoring (SRM) using the peak area. The transition ions were selected based on predominant fragmentation pathways of standards and their intensity, as observed in their product ion mass spectra. In addition, the use of the SRM transition of lorazepam-*d*_4_ at *m*/*z* 325.00 > 279.05 resulted in a significant decrease in selectivity due to the interference of isotope ions of chlorine derived from lorazepam. To improve selectivity, we used the transition at *m*/*z* 327.0 > 280.9 as described by Kamal et al. [15]. The optimized SRM transitions and retention times of compounds are summarized in Table 1. Data acquisition and processing were performed using LabSolutions software (v 5.99; Shimadzu).

**Table 1:** SRM transitions and parameters for detection of the analytes and internal standards

Analyte	Retention time (min)	Precursor (*m*/*z*)	Product (*m*/*z*)	Q1 (V)	CE (eV)	Q3 (V)
THC-COOH	4.213	**345.1**	**327.1**	** − 13**	− **16**	− **23**
			299.1	− 14	− 19	− 15
THC-COOH-*d*_9_	4.240	**354.2**	**336.1**	− **14**	− **17**	− **24**
			308.2	− 14	− 20	− 23
THC-COOH glucuronide	3.790	**521.4**	**345.1**	− **20**	− **14**	− **25**
			327.1	− 40	− 24	− 23
Oxazepam	2.796	**286.9**	**240.9**	− **11**	− **23**	− **26**
			268.9	− 12	− 15	− 19
Oxazepam-*d*_5_	2.815	**291.9**	**246.0**	− **12**	− **21**	− **25**
			273.9	− 11	− 16	− 20
Oxazepam glucuronide	2.246	**463.1**	**287.0**	− **18**	− **15**	− **21**
			241.0	− 18	− 36	− 26
Lorazepam	2.838	**321.0**	**274.9**	− **13**	− **22**	− **19**
			302.9	− 13	− 16	− 22
Lorazepam-*d*_4_	2.823	**327.0**	**280.9**	− **14**	− **24**	− **30**
			309.0	− 13	− 16	− 22
Lorazepam glucuronide	2.335	**497.2**	**320.9**	− **20**	− **14**	− **24**
			274.9	− 20	− 37	− 29
Temazepam	2.961	**301.0**	**255.0**	− **11**	− **21**	− **18**
			283.0	− 12	− 13	− 21
Temazepam-*d*_5_	2.945	**306.0**	**260.1**	− **13**	− **23**	− **27**
			288.0	− 11	− 15	− 14
Temazepam glucuronide	2.451	**477.2**	**255.0**	− **12**	− **34**	− **28**
			301.0	− 11	− 14	− 22
Amitriptyline	2.830	**278.1**	**233.0**	− **11**	− **16**	− **16**
			91.0	− 11	− 26	− 17
Amitriptyline-*d*_3_	2.829	**281.1**	**233.0**	− **11**	− **17**	− **16**
			105.0	− 11	− 23	− 19
Amitriptyline glucuronide	2.732	**454.2**	**233.1**	− **17**	− **21**	− **24**
			278.1	− 13	− 22	− 29

The quantitative transitions are shown in bold type

*CE* collision energy

### Method validation

All validation studies were performed using NHU according to the acceptance criteria of the Ministry of Health, Labour and Welfare Guideline on Bioanalytical Method Validation in Pharmaceutical Development and related documents [16, 17].

### Selectivity

The selectivity of the entire analytical procedure was verified by analyzing blank samples (*n* = 6) under optimized chromatographic conditions. At the same retention time as that of the quantitative transition, the absence of any chromatographic signal (signal-to-noise ratio (S/N) ratio > 3) indicated no interference of endogenous substances.

### Calibration curve and linearity

Calibration curves were established with seven points by plotting the peak area ratios. Calibrator samples were freshly prepared in NHU at concentrations of 0.5, 5, 10, 50, 100, 250, and 500 ng/mL for amitriptyline and temazepam; at concentrations of 2, 10, 20, 100, 200, 500, and 1000 ng/mL for lorazepam and oxazepam; and at concentrations of 5, 10, 20, 100, 200, 500 and 1000 ng/mL for THC-COOH. Samples of each concentration were injected in triplicate, and six independent replicates per concentration were analyzed. Calibration curves were generated using a least squares linear regression with an inverse-variance weighting factor in W-REG V2.2 software, which is freely available on the website (https://www2u.biglobe.ne.jp/SATORU/).

### Limits of detection and quantification

The lower limit of quantification (LLOQ) was defined as the lowest point of the calibration curve that had an S/N of greater than 10:1. The lower limit of detection (LLOD) was determined as the lowest concentration yielding an S/N of 3:1.

### Intra-day and inter-day accuracy and precision

The intra- and inter-day accuracies and precisions were assayed at four concentration levels of QC samples (LLOQ, low, medium, and high) in the linear dynamic range by analyzing six replicates on 6 different days. The accuracy of the assay was expressed by comparing the calculated concentrations of QC samples to their respective nominal values × 100%. Precision was expressed as the coefficient of variation. The criteria for acceptable accuracy and precision were set at within 20% for the LLOQ and 15% for other concentrations.

### Recovery efficiency and matrix effect

Recovery efficiencies and matrix effects were determined at three concentration levels of QC samples (low, medium, and high) and calculated by comparing peak areas. Three sets of samples were prepared: NHU extracts with the analytes spiked before extraction (A); NHU extracts with the analytes spiked after extraction (B); and standard solutions at equivalent concentrations (C). The recovery efficiency and matrix effect were expressed as A/B × 100% and B/C × 100%, respectively.

### Carry-over

Carry-over was evaluated by analyzing a blank sample following analysis of the highest concentration calibration standard. No evidence of carry-over was observed.

### Stability

The short-term stability of the glucuronide conjugates was conducted by performing LC–MS/MS analyses of samples after incubating two concentrations of spiked urine at room temperature for 4 h. Long-term stability was assayed following incubation of spiked urine samples at − 30 °C or − 80 °C for 28 days (d). The stability of the compounds during freeze–thaw cycles was determined following three cycles of thawing spiked urine samples at room temperature and then refreezing at − 30 °C for a minimum of 12 h. All samples were analyzed after hydrolysis as described in the “Sample extraction” section.

### Application of the method to postmortem urine samples

The established method was tested for applicability and validity by using it to analyze 12 postmortem urine samples. Urine samples were obtained during autopsies performed in our laboratory from 2013 to 2022 and stored at − 80 °C until analysis. Urine samples were selected based on positive results in previous routine toxicological analyses performed by LC–MS/MS. Urine samples with concentrations higher than the upper limit of quantification were appropriately diluted before determination.

## Results

### Optimization of hydrolysis conditions

**Fig. 1:**
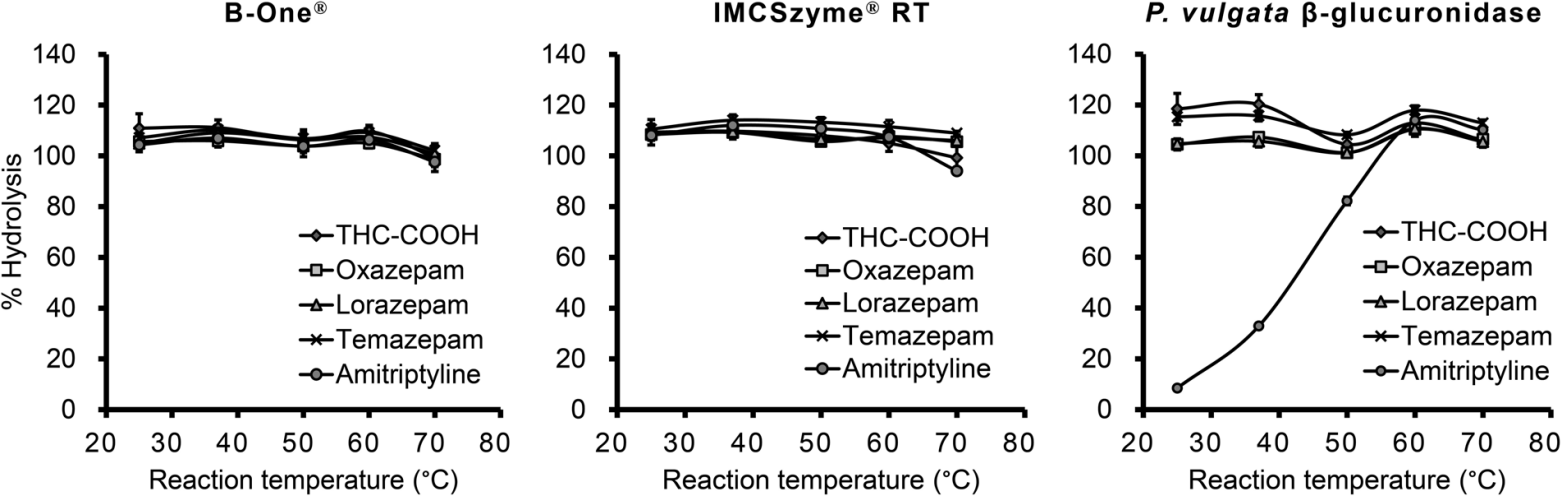
Effect of reaction temperature on the hydrolysis of the glucuronides of five psychoactive drugs by three β-glucuronidase preparations (*n* = 3)

First, the temperature dependences of the glucuronide conjugate hydrolysis activities of three commercially available enzymes (B-One®, IMCSzyme® RT and *P. vulgata* β-glucuronidase) were examined. The reaction times and enzyme volumes were set according to the instructions supplied by the manufacturers of the enzymes as follows: reactions with B-One® included 7200 U of the enzyme solution and proceeded for 5 min, reactions with IMCSzyme® RT included 1000 U of the enzyme and proceeded for 15 min, and reactions with *P. vulgata* β-glucuronidase included 2000 U of the enzyme and proceeded for 60 min. After the hydrolysis reactions, the samples were loaded onto extraction columns to remove the enzymes and any biological matrix. As shown in Fig. 1, B-One® and IMCSzyme® RT achieved complete hydrolysis of five glucuronide conjugates at 25 °C (room temperature), whereas *P. vulgata* β-glucuronidase required temperatures higher than 60 °C to completely hydrolyze amitriptyline glucuronide. Therefore, we selected B-One® and IMCSzyme® RT for further optimization.

**Fig. 2:**
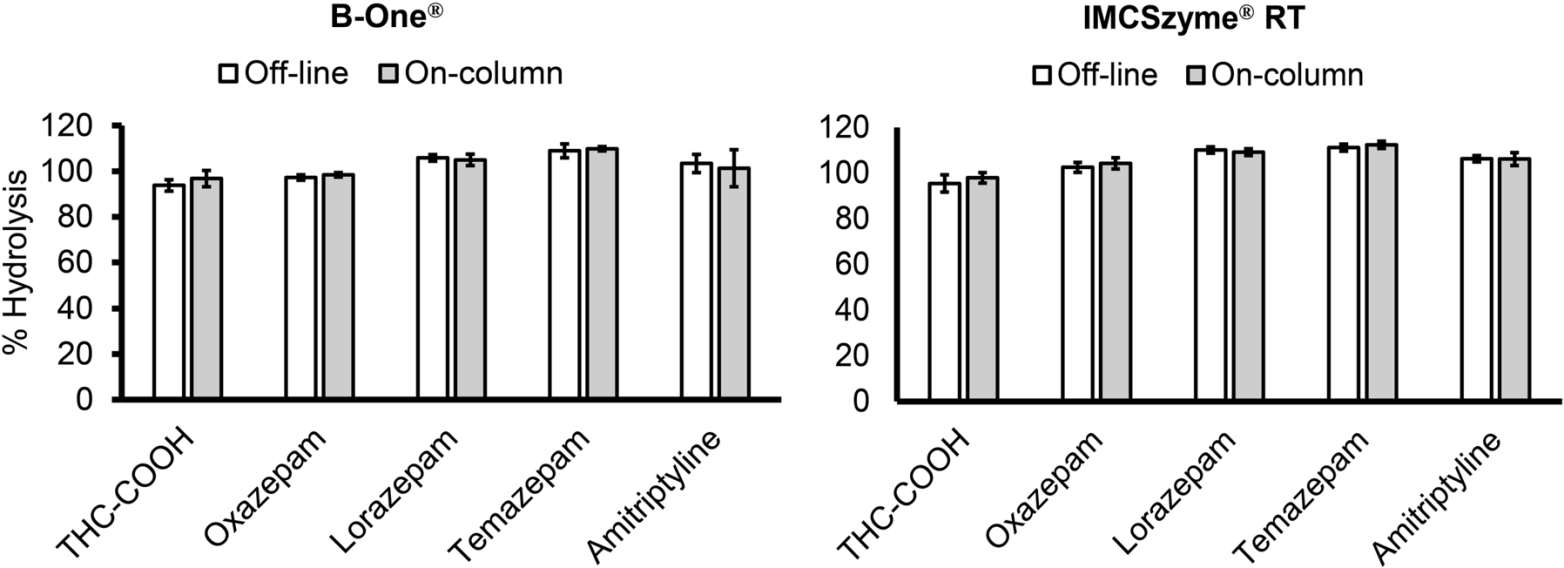
Comparison of the off-line and on-column hydrolysis efficiencies of two commercially available recombinant β-glucuronidase preparations (*n* = 3). Spiked urine samples were either subjected to hydrolysis and then extracted with extraction columns (off-column) or subjected to hydrolysis on the extraction columns (on-column)

Since B-One® and IMCSzyme® RT were found to be active at room temperature, in these subsequent experiments, mixtures of spiked urine samples and hydrolyzing enzymes were loaded directly onto extraction columns, and hydrolysis was allowed to proceed on-column. The on-column reactions proceeded with the same efficiency of hydrolysis as observed with the off-column method, in which the samples were loaded onto the extraction column after the hydrolysis reactions (Fig. 2).

**Fig. 3:**
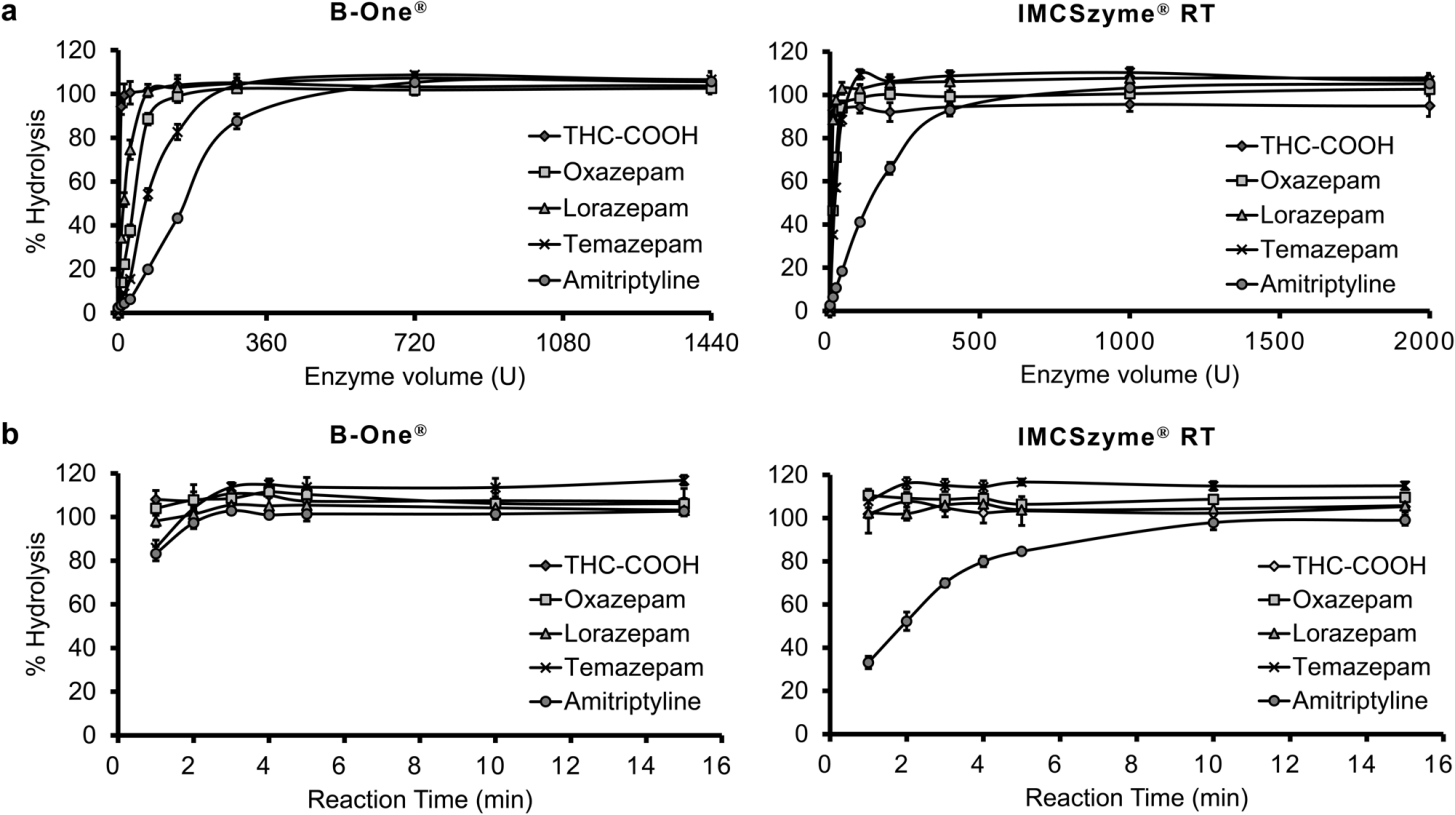
Optimizing the hydrolysis of drug glucuronides by recombinant β-glucuronidases (*n* = 3). Effect of (**a**) enzyme volume and (**b**) reaction time on the hydrolysis of the glucuronides of five psychoactive drugs with B-One® and IMCSzyme® RT

The on-column, room temperature hydrolysis of the five conjugates by B-One® and IMCSzyme® RT was further optimized by investigating the effects of enzyme volume and reaction time. As shown in Fig. 3a, ≥ 720 U of B-One® was required to completely hydrolyze all five glucuronide conjugates during 5 min reactions at room temperature. More than 1000 U of IMCSzyme® RT was required to completely hydrolyze all five glucuronide conjugates during 15 min reactions at room temperature (Fig. 3a). Under these conditions, B-One® was found to completely hydrolyze the five glucuronide conjugates within 3 min (Fig. 3b). The hydrolysis of four of the glucuronide conjugates by IMCSzyme® RT was similarly rapid, but this enzyme required more than 10 min to completely hydrolyze the amitriptyline conjugate (Fig. 3b). Because of the more consistent rate of hydrolysis by B-One®, we focused on this enzyme in subsequent validation assays.

### Validation of the method

The enzyme-based method to quantify drug conjugates in urine was evaluated by determining the calibration curve range, regression equations, coefficient of determination (*r*^2^), LLOD, and LLOQ values (Table 2). The calibration curves demonstrated good linear relationships over the range of drug concentrations tested, with *r*^2^ values exceeding 0.998.

**Table 2:** Linearity, limit of detection (LLOD) and limit of quantification (LLOQ)

Analyte	Range (ng/mL)	Regression equation	Coefficient of determination (*r*^2^)	LLOD (ng/mL)	LLOQ (ng/mL)
THC-COOH	5–1000	*y* = 0.00675*x* − 0.00243	0.9986	2	5
Oxazepam	2–1000	*y* = 0.00530 *x* − 0.00028	0.9995	1	2
Lorazepam	2–1000	*y* = 0.00843*x* − 0.00031	0.9977	0.5	2
Temazepam	0.5–500	*y* = 0.00788*x* − 0.00010	0.9979	0.1	0.5
Amitriptyline	0.5–500	*y* = 0.00807*x* − 0.00002	0.9988	0.1	0.5

The intra- and inter-day accuracies and precisions were evaluated at four concentration levels (Table 3). The accuracies ranged from 93.0% to 109.7%, and the precisions ranged from 0.8% to 8.8%. These values were considered acceptable at all concentrations. The recovery efficiencies ranged from 56.1% to 104.5%, while the matrix effects ranged from 78.9% to 126.9% (Table 3).

**Table 3:** Method validation: intra- and inter-day accuracies and precisions, recovery efficiencies and matrix effects

Analyte	QC concentration (ng/mL)	Intra-day (*n* = 6)		Inter-day (*n* = 6)		Recovery efficiency ^a^ (%)	Matrix effect ^a^ (%)
		Accuracy (%)	Precision (CV, %)	Accuracy (%)	Precision (CV, %)		
THC-COOH	5	101.8	2.8	105.2	4.5	-	-
	15	98.1	2.7	103.1	6.7	56.1 ± 2.8	109.2 ± 3.0
	150	99.5	1.6	102.3	3.7	57.2 ± 4.1	93.9 ± 3.7
	800	101.1	2.5	102.3	3.5	60.4 ± 2.3	80.8 ± 3.7
Oxazepam	2	101.9	2.5	93.0	5.8	-	-
	3	105.6	2.9	94.5	8.3	66.4 ± 3.6	99.9 ± 1.1
	150	107.8	0.8	98.4	8.8	74.8 ± 3.8	100.1 ± 3.5
	800	109.7	1.5	99.0	6.2	79.6 ± 1.5	86.3 ± 4.6
Lorazepam	2	99.9	3.1	100.9	6.7	-	-
	3	101.0	3.0	102.4	3.4	70.2 ± 4.3	100.7 ± 2.4
	150	96.5	1.0	95.8	6.0	76.8 ± 2.9	126.9 ± 3.5
	800	96.0	2.2	97.4	4.2	80.1 ± 1.7	93.5 ± 6.2
Temazepam	0.5	99.9	1.5	103.5	5.7	-	-
	1.5	99.3	2.1	102.3	3.5	75.3 ± 2.4	102.2 ± 1.7
	75	95.5	1.3	98.2	2.4	84.2 ± 2.3	99.9 ± 3.9
	400	99.0	1.2	102.6	1.9	85.4 ± 3.4	88.5 ± 3.6
Amitriptyline	0.5	102.4	2.5	100.6	3.6	-	-
	1.5	100.5	1.9	99.9	2.5	92.6 ± 2.0	93.7 ± 4.8
	75	93.7	1.3	93.2	2.0	102.7 ± 2.9	82.5 ± 2.3
	400	100.1	1.9	100.4	2.7	104.5 ± 4.6	78.9 ± 3.7

^a^Values are expressed as the mean ± SD (*n* = 6)

The stabilities of glucuronide conjugates during storage within the biological matrix were evaluated by determining the accuracy of detection after storage of spiked urine samples under different conditions (Table 4). The drug glucuronides were all found to be acceptably stable under all tested conditions, except for THC-COOH glucuronide, which exhibited relatively low stability during long-term storage and freeze–thaw cycles.

**Table 4:** Stability of analyte glucuronide conjugates in urine under different storage conditions

Analyte	QC concentration (ng/mL)	Short-term (4 h), room temperature (%) (*n* = 6)	Long-term (28 d), − 30 °C (%) (*n* = 6)	Long-term (28 d), − 80 °C (%) (*n* = 6)	Freeze–thaw, three cycles (%) (*n* = 6)
THC-COOH glucuronide	25	98.2	68.2	74.5	64.1
	1200	102.4	60.6	64.2	71.4
Oxazepam glucuronide	5	100.0	104.1	107.7	88.2
	1200	96.3	94.0	92.6	86.4
Lorazepam glucuronide	5	95.7	94.1	92.8	85.6
	1200	104.5	102.1	103.7	94.3
Temazepam glucuronide	3	90.4	94.1	101.1	81.0
	600	96.5	92.4	90.3	86.5
Amitriptyline glucuronide	3	93.6	99.3	96.2	92.1
	600	109.8	108.5	112.8	104.4

### Application of the method to postmortem urine samples

**Fig. 4:**
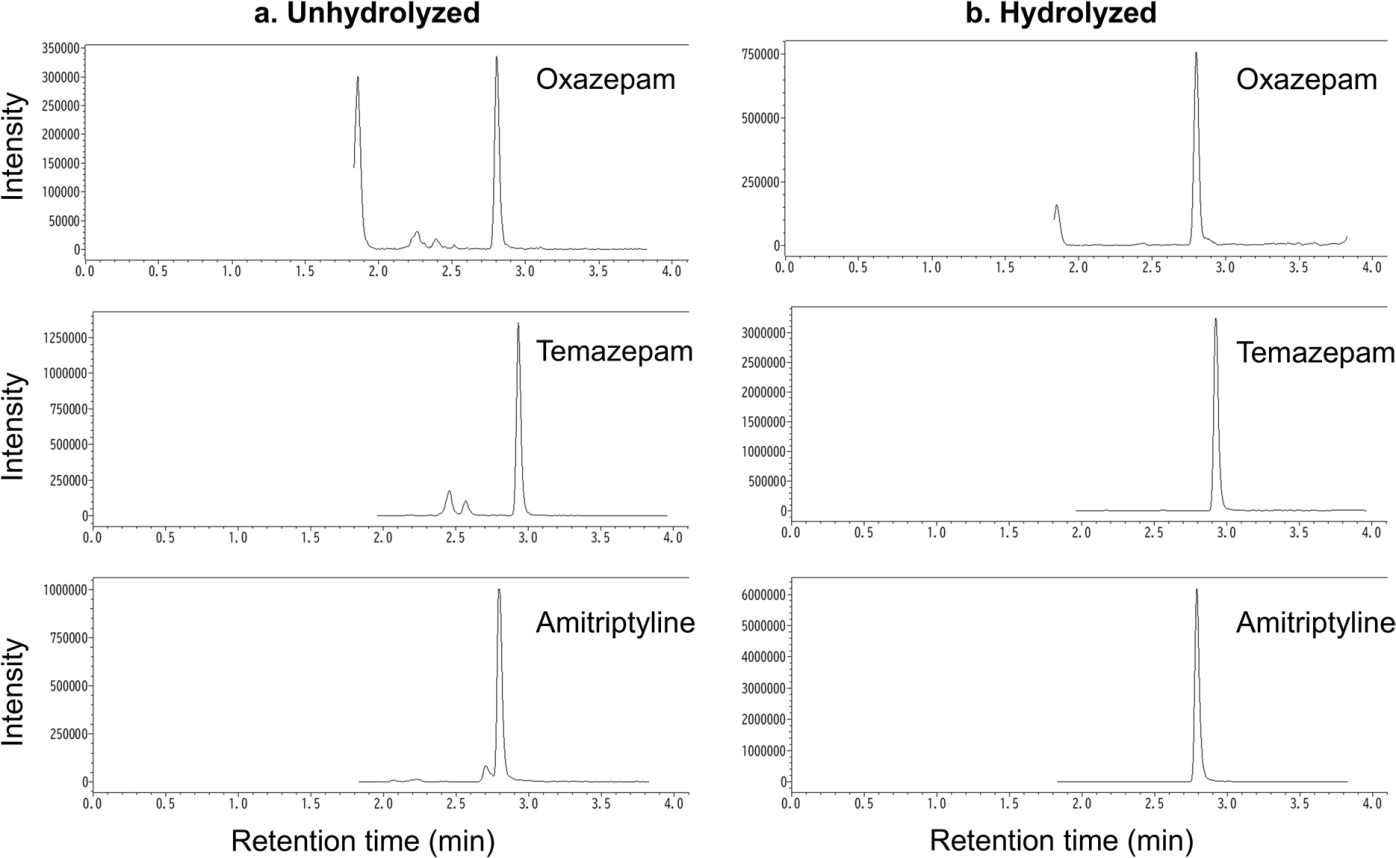
Representative SRM chromatograms of extracts from (**a**) unhydrolyzed and (**b**) hydrolyzed sample No. 4 in Table 5

After validation, the established method was applied to 12 authentic forensic samples of urine (Table 5). Representative SRM chromatograms of extracts from unhydrolyzed and hydrolyzed sample No. 4 in Table 5 are shown in Fig. 4. The peaks of the analytes were clearly observed with retention times within 5 min. There were no peaks that interfered with the measurements. In the present study, the glucuronide conjugates of the authentic forensic samples of postmortem urine were not quantified, so the exact binding levels could not be determined. However, the glucuronidation percentages were calculated for each individual sample from the concentration of the free drugs and metabolites analyzed before and after hydrolysis. The percent glucuronidation values obtained for each drug were as follows: THC-COOH (42.5–82.0%), oxazepam (93.1–99.7%), lorazepam (99.0–99.3%), temazepam (95.9–99.8%), and amitriptyline (82.2–96.0%).

**Table 5:** Quantification of psychoactive drugs in authentic forensic samples of postmortem urine using ISOLUTE HYDRO DME + column extraction

Sample No	Analyte	Unhydrolyzed (ng/mL)	Hydrolyzed (ng/mL)	Glucuronidation (%)^a^
1	THC-COOH	731.0	1270.7	42.5
2	THC-COOH	102.5	569.7	82.0
3	Amitriptyline	367.5	2060.7	82.2
4	Amitriptyline	649.2	16,102.4	96.0
	Oxazepam	416.3	6036.9	93.1
	Temazepam	287.8	6988.0	95.9
5	Oxazepam	22.3	1251.6	98.2
	Temazepam	11.0	794.7	98.6
6	Oxazepam	3.1	330.6	99.1
	Temazepam	6.0	576.1	99.0
7	Oxazepam	1.9	470.3	99.6
	Temazepam	0.5	180.3	99.7
8	Oxazepam	10.6	320.5	96.7
	Temazepam	3.5	113.2	96.9
9	Oxazepam	< LOQ	9.7	–
	Temazepam	4.1	127.4	96.8
10	Oxazepam	2.0	641.2	99.7
	Temazepam	0.5	235.0	99.8
	Lorazepam	2.7	416.1	99.3
11	Lorazepam	12.5	1306.4	99.0
12	Lorazepam	73.8	7662.1	99.0

^a^Glucuronidation (%) = (hydrolyzed analytes − unhydrolyzed analytes) / hydrolyzed analytes × 100

## Discussion

We have established an LC–MS/MS method for five psychoactive drugs (THC-COOH, oxazepam, lorazepam, temazepam and amitriptyline) in urine after enzymatic hydrolysis of glucuronide conjugates. The present study demonstrated that the recombinant β-glucuronidase B-One® was found to completely hydrolyze the five glucuronide conjugates within 3 min directly on extraction columns (Fig. 3b). The method was successfully applied to forensic autopsy samples after thorough validation.

The present method including enzymatic hydrolysis of glucuronide conjugates followed by LC–MS/MS analysis has several advantages. First, the recombinant β-glucuronidase B-One® quantitatively hydrolyzed glucuronide conjugates at room temperature, so hydrolysis could be performed directly on extraction columns. This on-column method does not require a transfer of the hydrolyzed sample to the extraction column; this more efficient extraction process saves time and eliminates the loss of valuable samples during transfer. The hydrolysis time of this method was shorter than those observed in previous studies [3–6]. The hydrolysis of four of the glucuronide conjugates by IMCSzyme® RT was similarly rapid, but this enzyme required more than 10 min to completely hydrolyze the amitriptyline conjugate. Amitriptyline glucuronide linked to quaternary ammonium compounds has been reported to be difficult to hydrolyze with β-glucuronidase [12, 13]. Second, the sensitivity of the analysis of target compounds was improved 1.7–470 times by hydrolyzing glucuronides as shown in Table 5, showing that urinary drug concentrations can be evaluated accurately.

The stability experiments showed that the drug glucuronides were acceptably stable under all tested conditions although THC-COOH glucuronide exhibited relatively low stability during long-term storage and freeze–thaw cycles (Table 4). Notably, acyl glucuronides, including THC-COOH glucuronide, have been shown to be generally unstable [18]; therefore, it is necessary to pay careful attention to the stability of THC-COOH glucuronide.

The total concentrations of THC-COOH determined in two of the postmortem urine samples (Table 5) were consistent with existing reports, which have documented postmortem urine concentration ranges of THC-COOH of 18–1562 ng/mL [19] and 24.2–970 ng/mL [20]. Similarly, the percent glucuronidation values identified by this method (Table 5) were comparable to those previously reported in patients’ urine [6–10] and in authentic forensic urine samples [21]. Importantly, these findings suggest that oxazepam and temazepam have high conjugation rates and that only trace amounts of the unconjugated target drugs are excreted in the urine. It may thus be preferable to hydrolyze any conjugates prior to urinalysis in which target medications are not provided.

## Conclusions

In this study, the commercially available recombinant β-glucuronidase B-One® was used to hydrolyze glucuronide conjugates of five psychoactive drugs (THC-COOH, oxazepam, lorazepam, temazepam, and amitriptyline) within 3 min at room temperature. In addition, the pretreatment process was shortened by hydrolyzing the glucuronide conjugates directly on the extraction columns. This analysis method was successfully applied to forensic autopsy samples after thorough validation. We expect that the method described in this study will have broad applications, including in clinical and forensic toxicological investigations.
